# 

**Published:** 1892-02-06

**Authors:** 


**Notices of Births, Deaths, and Marriages,** are inserted in this column at a charge of 2s. 6d. for an announcement not exceeding four lines.

### NOTICE TO CORRESPONDENTS.

All MS., Letters, Books for Review, and other matters intended for the Editor should be addressed The Editor, The Lodge, Porchester Square, London, W.
The Editor cannot undertake to return rejected M.S., even when accompanied by stamped directed envelope.

**Notice to Advertisers and Subscribers.**

All Advertisements, Orders for Copies of The Hospital, and Business Communications should be addressed to The Publisher, *not to the Editor*, The Hospital, 140, Strand, W.C.

The Cost of Subscription, post free, is as follows:—Per week, 1½d.; per quarter, 1s. 8d.; per half-year, 3s. 3d.; per annum, 6s. 6d. Foreign:—Per annum, 8s. 8d.; payable, in all cases, in advance.

**"Burdett's Hospital Annual," 1891—1892.**

Third year of publication. 600 pp. Boards, gilt lettering. A most complete guide to British and Colonial Hospitals, Dispensaries, Nursing and Convalescent Institutions, and Asylums. Price 3s. 6d. Published by The Hospital (Limited), 140, Strand, W.C.

**NOTICE.—The Index for Vol. X. (ending September, 1891), is on sale at the Office of this Journal, price 2½d., post free.**

### General Charities.

[This Column is devoted to an account of work of charitable institutions other than Medical Charities. The Editor will be glad to receive reports or news of work at 140, Strand. *Philanthropy* should be plainly written on the envelope.]

The Ladies' Charity School, Powis Gardens, W., founded at the beginning of the last century, is a most useful institution, and is the means each year of educating and training a number of girls whose parents are now in necessitous circumstances. Prize day was held on the 21st ult., when Lady Maria Ponsonby kindly distributed the awards, and an excellent programme of music and recitations, concluding with a musical drill remarkably well performed, was given. Colonel Anstruther Price, in introducing Lady Ponsonby, drew attention to the good done by the school under the management of Miss Wright; how the admission of the girls brought joy and comfort to the hearts of many struggling mothers, who felt that in the school their little ones had secured the comforts of a home in which the habits of thrift and order would be inculcated and good principles instilled. At the same time he impressed upon his audience that to keep the school up to its present state of efficiency to maintain and feed fifty-four girls, a considerable annual disbursement was necessary, and that, as the school did not advertise itself, the individual efforts of friends and subscribers formed the only means by which fresh funds could be obtained. We commend these words to the notice of our readers.

Lieut.-Col. J. B. Walker, late R.A., the indefatigable Hon. Secretary of the Church of England Soldiers' Institute, at Aldershot, has been selected as the commandant of the Gordon Boys' Home.

The annual meeting of the Children's Home and Orphanage was held at St. James's Hall on the 12th inst. Mr. J. Vanner presiding. There are 850 children in these homes, which were by Dr. Stephenson twenty years ago. The expenditure of the institutions amounts to £18,000 per annum, and this useful work is now £3,500 in debt.

The annual report of the Newspaper Press Fund shows that there are now 833 members, 105 having been added during the year. The present invested funds amount to £22,490, and the income during the year was £2,518, and £1,132 was spent in grants to 28 members and 34 non-members. The annual dinner will take place on May 14th, Mr. A. J. Balfour, M.P., presiding.

Mr. Henry Irving, who presided last week at the annual general meeting of **The Actors' Benevolent Fund**, was able to congratulate the members on a satisfactory year's work. The establishment of a number of local centres for the distribution of emergency relief has been attended with excellent results. But though year by year the great body of actors is drawing closer together, the happy consummation has still to be attained when every actor in the kingdom subscribes his mite for the support of his less fortunate brethren.

One of the oldest charitable institutions in the country is **The Christian Community** which is the outcome of an association, started as far back as 1685 by French refugees, for the religious teaching of the poor of London. The society as now constituted is purely unsectarian and ministers to the bodily wants of many toiling men whose hard and abject lives it endeavours to cheer, and whose poverty and want it strives to alleviate. The Treasurer, Mr. F. A. Bevan earnestly solicits support for the all night rescue work of the society, the poor children's dinner fund, the rescue of fallen women and placing them in homes, the working girls' home and institute, work in common lodging houses, lectures, concerts, and sewing classes, and other benevolent objects. During the seven years that the mission hall, in Thrall Street, Spitalfields, has been open, 34,000 men have found shelter from the cold and wet of winter nights. It appears that a great number of the men who seek the shelter either have no trade to take up, or have been taught a trade which is fast disappearing: This fact has been so impressed upon the Committee, that, if funds are forthcoming for the purpose, they hope to start some "trade classes" in order to give unskilled men an opportunity of learning something of a trade. We cordially wish them success in a direction which will enable such men to help themselves. Doles of food and lodging cannot prove of lasting use to any man; if carried too far such a system may help to pauperise; but money expended in the purchase of tools or clothing for those who are willing to work, but cannot, owing to the lack of these necessaries, will help a man permanently. This the society cannot always do as often as it would wish, owing to lack of funds.

### Scraps and Gleanings.

The Dublin Hospital Sunday Fund for 1891 amounted to £3,982 19s. 6d.

The Salters' Company have given a donation of 50 guineas to the Irish Distressed Ladies' Fund.

The Board of Management of the Liverpool Hospital for Women appeal for £3,000 to enlarge the hospital.

The total receipts of the "Leeds Workpeople's Hospital Fund" for the year 1891 amounted to the large sum of £5,621 19s. 11d.

We regret to learn that Mr. Harry Furniss is suffering from overwork, and has been ordered by his medical adviser complete rest for several weeks.

Lord Tennyson contributes to the February number of the *Nineteenth Century* a short poem of seventeen lines on the death of the Duke of Clarence.

The Countess of Aberdeen has opened at Glasgow a hospital, which is to be free to women only, the patients to be attended by qualified female practitioners.

Sir Oscar Clayton, C.M.G., F.R.S., the eminent surgeon, died on the 26th inst. He was born in London in 1816, and has passed away universally beloved and regretted.

It is proposed to make important alterations and extensions to Edinburgh Royal Infirmary. The estimated cost of the site and furnishings of the new buildings is £73,500.

The Court of Common Council has voted two sums of 100 guineas for the relief of the widows and families of the fishermen drowned in the recent gales off Great Yarmouth and Lowestoft.

Gardeners are notoriously a long-lived race, but James Williams, whose death was lately recorded in *The Times* beats the record. He wa 94 years old, and had lived 74 years in one place.

The portrait painted by Mr. Herkomer of the late Duke of Clarence will shortly be on view at the Fine Art Society. Messrs. Agnew are showing Mr. Dampier May's portrait of Cardinal Manning.

The Metropolitan Asylums Board have made arrangements for placing their ambulance service at the disposal of the public to facilitate the isolation of influenza patients in places other than the Board's hospitals.

Sir George Edward Paget, K.C.B., Regius Professor of Physic at Cambridge University, elder brother to Sir James Paget, the eminent surgeon, died at Cambridge on the 29th inst., in the 83rd year of his age.

Special features in the work of the London Vegetarion Society during the past year have been the practical demonstrations in halfpenny dinners for schools, fruit banquets, and addresses on food reforms in Board schools.

The entrance fees of the Benevolent Billiard Handicap amounting to £336, will be equally divided between the Bentinck Benevolent Fund, the Rous Memorial and Brompton Hospitals, and the Licensed Victuallers' Asylum.

The report of the Eastern Dispensary, Bath, states that with a balance from last year of £123 13s. 9d., the receipts amounted to £817 0s. 5d., and the expenditure to £687 0s. 6d., leaving a balance in hand of £129 19s. 11d.

The money raised at Harrowgate by a ladies' committee to purchase a wedding present for the late Duke of Clarence is to be devoted to founding a ward in the cottage hospital, to be called the Albert Victor or Clarence Ward.

The *tableaux vivants* for the benefit of the poor of Chelsea, which were to have taken place at the town hall on Tuesday, 26th inst., have been postponed until February 29th. Numerous members of the Royal Family have given their patronage.

The male students of the American Medical College of St. Louis, United States, have been in revolt against the formidable rivalry of the women students. The Dean has called upon all the signatories to withdraw the petition or withdraw themselves from the College.

The balance-sheet for 1891 of the West Kent General Hospital showed a balance in hand of £207 9s. 5d. The receipts amounted to £7,327 0s. 1d. and the expenditure, including the adverse balance of £98 6s. 8d. with which the year was begun, amounted to £7,119 10s. 8d.

The Misses Stokes have promised, as a memorial to their brother, the late Mr. Alfred Stokes, the sum of £7,500 for the purchase of Tyn-y-Coed, Llanrhos, near Llandudno, for the purpose of a convalescent home in connection with the Birmingham Hospital Saturday Fund.

The annual report of the Newton Cottage Hospital showed that the receipts for the past year amounted to £532 12s. 7d., and the expenditure to £507 12s. 3d., leaving a balance in hand of £25 0s. 4d. The number to patients treated (120) is considerably in excess of any former year.

The directors of the Aberdeen Hospital for Incurables have issued a special appeal for funds to cancel the debt incurred in recent necessary additions to that admirable institution, of which Dr. Urquhart and Dr. G. E. Cooper are the medical officers, and Mr. P. Cooper the secretary.

Orders have been received from Her Majesty's Government that the police at Cape Town, and the Government Hospitals at Malta, shall be supplied with Mr. Alfred Carter's (17, Holborn Viaduct) Street Ambulance.

"We are able to state," says the *British Medical Journal*, "that the President of the Royal College of Physicians is in communication with the President of the Local Government Board on the suggested appointment of a Royal Commission for inquiry into the causation, prevention, &c., of influenza."

The late Mr. John Palmer Stocker, of Oxford Terrace, Hyde Park, has left £1,000 to the Medical Benevolent College, Epsom; £200 to the Truss Society, Finsbury Square; £100 to the Great Ormond Street Hospital for Sick Children; £100 to St. Mary's Hospital, Paddington; £100 to the St. Andrew's Ladies' Lying-in Charity; £100 to the Cancer Hospital, Brompton; and various sums to other charities.

According to the Registrar-General's returns the mortality in London for the week ending January 23rd was at the rate of 46 per 1,000 more than double the normal proportion; 506 deaths from influenza were reported as compared with 271 in the preceding seven days. The death-rate of Brighton was still higher, 60 per 1,000, but then the population of Brighton normally comprises a very large proportion of invalids.

### THE STUDENT OF MEDICINE.

[All communications for this Supplement should be addressed to THE EDITOR of THE HOSPITAL, at 140, Strand, with the words, "Students' Column," written in the left hand top corner of the envelope.]

### APPOINTMENTS.

Dudley W. Buxton, M.D., M.R.C.P., appointed Anæsthetist to the National Hospital for the Paralysed and Epileptic, Queen Square, vice Dr. J. F. Silk, resigned. E. Tenison Collins, M.B., C.M., appointed Demonstrator of Anatomy to the University College, Dundee. E. Treacher Collins, F.R.C.S.Eng., appointed Ophthalmic Surgeon to the Belgrave Children's Hospital. H. G. G. Cook, M.B., B.S., F.R.C.S., appointed House Surgeon to the Victoria Hospital for Sick Children, vice Robert Nairn, M.R.C.S., L.R.C.P. H. Fraser, M.A., M.B., C.M.Aberd., appointed House Surgeon to the Edinburgh Royal Maternity and Simpson Memorial Infirmary. C. Brooke Gratte, M.R.C.S., L.R.C.P.Lond., appointed Honorary Surgeon to the Newport and County Infirmary. Richard Henry Grimbly, M.R.C.S.Eng., L.S.A., reappointed Surgeon to the Newton-Abbott Cottage Hospital and Dispensary. R. J. Hamilton, M.R.C.S., appointed Assistant Honorary Ophthalmic Surgeon to the Stanley Hospital, Liverpool. D. B. Hart, M.D.Edin., F.R.C.P., appointed Physician to the Edinburgh Royal Maternity and Simpson Memorial Hospital. Edgar Haydon, M.B., C.M.Glas., reappointed Surgeon to the Newton-Abbott Cottage Hospital and Dispensary. Dr. Jackson appointed House Surgeon to the South Charity Infirmary, Cork, vice Dr. N. Runciman, resigned. G. D. B. Levick, M.R.C.S., L.R.C.P., L.S.A., appointed Obstetric House Physician at the Middlesex Hospital. J. N. Lewis, M.R.C.S., L.R.C.P.Lond., appointed Honorary Surgeon to the Newport and County Infirmary. John William Ley, F.R.C.S.Eng., L.S.A., reappointed Surgeon to the Newton-Abbott Cottage Hospital and Dispensary. R. McLay, M.B., C.M.Glas., appointed Visiting Surgeon to the Dumbartonshire Combination Fever Hospital. Hugh M. Montgomerie, M.D., M.B., C.M.Edin., appointed Physician to the West Cornwall Infirmary and Dispensary, vice J. Barclay Montgomery, L.R.C.P., L.R.C.S., resigned. James Barclay Montgomery, L.R.C.P., L.R.C.S., appointed Consulting Physician to the West Cornwall Infirmary and Dispensary. H. O. Nicholson, M.B., C.M.Aberd., appointed House Physician to the Aberdeen Hospital for Sick Children. William Gifford Scott, M.B., C.M., L.R.C.S.Edin., reappointed Surgeon to the Newton-Abbott Cottage Hospital and Dispensary. John Symons, M.R.C.S.Eng., L.S.A., reappointed Surgeon to the West Cornwall Infirmary and Dispensary. Reginald Robert Whishaw, B.A., M.B., B.C.Cantab., F.R.C.S.Eng., L.R.C.P.Lond., appointed Surgeon to the Croydon General Hospital, vice Dr. Lister, resigned. Thomas Wilmot, L.R.C.P.Lond., M.R.C.S., appointed Visiting Physician to the Bradford Fever Hospital, vice Samuel Brown, M.D. H. H. Du Boulay, M.R.C.S., and Walter Burt, L.D.S., M.R.C.S.Eng., have been appointed, the former as Assistant Honorary Medical Officer, the latter as Honorary Surgeon-Dentist, to the Royal Hospital and Dispensary, Weymouth.

### VACANCIES.

### Resident Medical Officers.

Borough Lunatic Asylum, Portsmouth, Clinical Assistant for six months—Board, &c.

Bucks County Asylum, Stone, near Aylesbury, *Locum tenens* for one month certain—Remuneration offered from £2 2s. per week, according to asylum experience.

Coppice Asylum, Nottingham, Assistant Medical Officer, unmarried, and not more than 28 years of age—Salary £120 per annum, board, &c.

East London Hospital for Children, Shadwell, E., Medical Officer, unmarried, appointment for two years—Salary £80 per annum, with board, &c.

East London Hospital for Children, Shadwell, E., House Physician—Board, &c.

Glamorganshire and Monmouthshire Infirmary and Dispensary, Cardiff, House Surgeon, doubly qualified—Salary £100 per annum, board, &c.

Hospital for Consumption and Diseases of the Chest, Brompton, House Physicians.

Hospital for Women, Soho Square, W., House Physician, doubly qualified, appointment for six months—Salary, £30.

Kent County Lunatic Asylum, Chartham, near Canterbury, Medical Superintendent—Salary £600 per annum, house, &c.

Nottingham General Hospital, Medical Assistant, appointment for six months—Board, &c.

Radcliffe Infirmary, Oxford, House Surgeon, doubly qualified—Salary £80 per annum, with board, &c.

### Non-Resident Medical Officers.

Dental Hospital of London, Medical Tutor—Salary £40 per annum. Applications to the Dean by February 8th.

London Lock Hospital and Asylum, Surgeon to in-patients and out-patients at 91, Dean Street, Soho.

Queen's Hospital, Birmingham, Casualty Surgeon—Honorarium £50 per annum.

